# On the Practical Study of Botany, by Means of Chemical Analysis:—Chiefly Abridged from the Original Papers of Dr. T. S. Hermbstaedt, &c.

**Published:** 1799-07

**Authors:** 


### *On the Practical Study of Botany, by means of Chemical Analyſis:—Chiefly abridged from the Original Papers of Dr.* T. S. Hermbstaedt, *&c.*

[Concluded from our last Number, p. 377 to 381.]

### *On the Proportion of Watery Parts in Vegetable Subſtances.*

In the chemical analyſis of vegetable bodies, we ought previouſly to attend to the circumſtance of their being completely freed from all humidity. To aſcertain this, let us weigh a determinate quantity, for inſtance, one hundred arbitrary parts of the ſubſtance under inquiry, whether this be an herb, a root, bark, flower, or wood.

The queſtion here is, of ſuch vegetables as have been previouſly dried in the air, and deprived of the greateſt portion of their humidity. If the experiment is to be made with green vegetables, we may chooſe the recently expreſſed juice for the analyſis; but in this caſe likewiſe, an accurate knowledge of the quantity of water it contains will be indiſpenſably neceſſary. Hence it will be proper to dry a certain quantity of the freſh herbs, aſcertained by weight, in order to learn howmuch they have loſt of their aqueous parts. By this comparative eſtimate, we ſhall be enabled to determine with tolerable preciſion, the relative proportion between vegetables in a freſh and a dry ſtate.

After

After theſe arrangements, a ſmall portion of the vegetable ſubſtance is put into a well-coated retort, with a ſimilar receiver, and the whole is ſubmitted for two hours, to what is called the water-bath. Although an experienced operator may employ the ſand-bath with equal advantage, by a very careful management of the fire, and the uſe of a good thermometer, by which he can manage the degree of heat, ſo as not to exceed the boiling point; yet the former proceſs is incomparably ſafer, inaſmuch as it will effectually prevent too high a degree of temperature, as well as the decompoſition of the vegetable matter under examination.

Having attended to theſe particulars during the operation, there will be uſually found in the receiver a ſmall quantity of a liquid, which, if the diſtilled ſubſtance was of an odoriferous nature, will be generally mixed with ſome eſſential oil. Meanwhile, we ſhall find in the retort the reſiduum of the diſtilled ſubſtance, in a ſtate of abſolute dryneſs, in which all other conſtituent parts remain unchanged, but deprived of their liquid. If both the receiver and the retort have been properly coated previous to the operation, the quantity of the liquid paſſed over in diſtillation will be evident from the increaſe of weight.

After removing the dry reſiduum, it ſometimes happens that the retort alſo has gained an acceſſion of weight, which is generally owing to ſome particles of ethereal oil having remained attached to its ſides.

If the ſubſtance thus diſtilled was deſtitute of odour, the liquor obtained from it may be conſidered in the analyſis as pure water. But if the ſubſtance contained an eſſential oil, or was combined with other volatile and fragrant particles, then the water in the receiver is either impregnated with odour, or the eſſential oil itſelf ſwims on the top. In the former caſe, the volatile and fragrant matter conſiſts either of pungent (acrid) or narcotic parts, which deſerve farther inveſtigation. In the latter caſe, the oily parts may be ſeparated by means of cotton, and their weight deducted from that of the whole fluid maſs. It deſerves, however, to be remarked, that a ſmall quantity, perhaps the 80th, or only 100th part of the oil remains diſſolved in the fluid reſiduum.

*On the ſeparation of oleaginous matter, or ethereal oil, from vegetables.*

If a vegetable ſubſtance, after previous examination, is ſuppoſed to contain any conſiderable quantity of eſſential oil, the following proceſs may be adopted for aſcertaining it by experiment. In the firſt place, we muſt procure ſeveral quarts of a ſtrong diſtilled water, from the ſame ſubſtance. Another quantity of that ſubſtance, not leſs than one pound weight, muſt be cut in ſmall pieces, and put into a retort with ſix times its weight of the diſtilled diſtilled water. Two thirds of this fluid are now to be drawn off by diſtillation, which is moſt advantageouſly performed in the ſand-bath. The oily parts thus ſeparated will be found in the fluid paſſed over into the receiver: and, as the water here employed was previouſly impregnated with as great a portion of ethereal oil as it was capable of holding in ſolution, the quantity of the oil obtained by this proceſs, may be conſidered as the true proportion exiſting in a given weight of the plant.

Theſe oleaginous particles frequently appear under different forms: they commonly either ſwim on the water, or ſink to the bottom, on account of their greater ſpecific gravity, which is particularly the caſe with the aromatic productions of hot climates; or, laſtly, they appear ſo intimately blended with the water, that it aſſumes an opaque, milky appearance. In the laſt caſe, the produce of diſtillation ought to be kept quietly, in a cloſe glaſs veſſel, for ſeveral days or even weeks: during which time the liquor generally becomes tranſparent, and the oily parts will be found adhering to the ſides, or bottom of the veſſel. Such oil, being in a manner cryſtallized, may be eaſily melted by heat, and poured out of the veſſel.

*On the method of obtaining camphor.*

Some vegetable ſubſtances, beſides the eſſential oil, contain likewiſe a portion of camphor, as one of their primary conſtituent parts. This ingredient may ſometimes be diſcovered by the ſmell, and the cooling, pungent taſte of ſuch ſubſtances; for inſtance, in the peppermint, cardamoms, &c. Such being the caſe, we may juſtly conjecture, that ſome camphor will be diſengaged by diſtillation, either in its pure ſtate, or in combination with eſſential oil. From this it may again be ſeparated, but not without a ſmall loſs of both, in the manner following: Diſſolve the whole of the camphorated oil, thus obtained, in a proportionate quantity of alkohol; which is capable of combining with the camphor as well as the eſſential oil. Dilute this ſolution with twelve parts of diſtilled water: if the oil contained no camphor, the water uſed for the mixture will remain clear and tranſparent; the alkohol will gradually combine with it, and the oily particles ſeparate. If, on the contrary, the oil was mixed with camphor, the whole of that fluid, on mixing it with water, will be converted into milky liquor, from which the camphor will gradually be precipitated in the form of a white powder, while the oleaginous parts aſcend to the ſurface of the water. By melting this powder in a cloſe glaſs veſſel, over a gentle fire, it will acquire the concrete form of camphor. The remaining liquor, however, will always retain ſome ſmall portion of the oil as well as the camphor.

On

*On the proceſs for ſeparating gum.*

As vegetable ſubſtances ſeldom, if ever, contain gum in a pure ſtate (for this is almoſt uniformly mixed with mucilage, ſoap, and reſin), it will be neceſſary, in the analyſis we undertake, to pay proper attention to theſe circumſtances.

Having previouſly aſcertained the exiſtence of gum in a vegetable body, we may proceed to analyſe it in the following manner:

1. A determinate ſmall quantity of the ſubſtance under experiment, according to weight, ſhould be placed in a glaſs veſſel, provided with a neck and receiver: four or ſix times the ſame quantity of the ſtrongeſt alcohol, by weight, muſt be added to the vegetable; and the whole kept for eight hours in tolerably ſtrong digeſtion.

2. After this, the liquor, as well as the whole of the macerated ſubſtance, muſt be ſtrained through a linen cloth. The reſiduum muſt then be again digeſted, for the ſame length of time, and with a ſimilar proportion of alkohol; after which, the extract is to be treated in the ſame manner as before.

3. As we may now be aſſured, that all reſinous and ſaponaceous matter, if ſuch exiſted in the ſubſtance, have been diſſolved in the alkohol, we ſhall find in the extracted reſiduum only the fibrous or woody parts, with the gum, and not unfrequently ſome mucilage.

4. In order to ſeparate thoſe parts, diſtilled water is to be poured on the reſiduum, and ſtrongly boiled with it in a tin kettle. The fluid part is afterwards poured off, freſh water is added, and the decoctions, are repeated, until the water manifeſt neither taſte nor colour, and the remaining vegetable fibre be likewiſe deprived of all ſapidity.

5. This operation being finiſhed, the remaining vegetable fibre is preſſed through a ſmall linen bag, which ought to be previouſly weighed; and after the bag and its contents have been completely dried, we ſhall find, from its ſubſequent weight, nearly the exact quantity of all ſoluble conſtituent parts which have been extracted during the operation.

6. The whole of the watery decoction thus obtained, after being properly filtered, ſhould be ſtrongly concentrated by ſlow evaporation, and afterwards ſuffered to ſtand quietly in a glaſs veſſel, ſlightly covered. If, during this time, ſome pulverulent matter ſhould fall to the bottom, it uſually conſiſts of reſinous particles, and ought to be preſerved, together with the ſpirituous extract.

7. If,

7. If, on the contrary, after ſeveral days, the liquor ſhould ſtill appear tranſparent, it then ought to be evaporated to complete dryneſs, in a glaſs veſſel (previouſly weighed), over a very gentle fire: in this caſe, the increaſe of weight in the veſſel will point out the quantity of the ſubſtance obtained.

8. If the extract was pure gum, the whole will be tranſparent, vitreous, and of an uneven fracture: but if it, at the ſame time, was mixed with mucilaginous matter, the inſpiſſated maſs will preſent a horny and ſemi-pellucid appearance, and have a granulated fracture.

*On the Separation of Reſinous Matter.*

If a plant ſubmitted to analyzation contained *gum*, it will be found either in a ſeparate ſtate, or combined with ſaponaceous matter. In order to ſeparate theſe two conſtituents of vegetable bodies from each other, they muſt be treated throughout as has been ſtated in the preceding ſection, relative to the ſeparation of gum; by which means we may obtain the reſin and ſoap, in a combined ſtate, diſſolved in the alcohol.—In order to ſeparate them effectually from each other, we proceed in the following manner:

1. The ſpirituous extract is poured into a retort, with the addition of the fourth part of diſtilled water: this mixture is ſubmitted to diſtillation, as long as a drop of ſpirituous liquor paſſes over into the receiver.

2. After this diſtillation is finiſhed, we ſhall find in the reſiduum reſinous matter, mixed, perhaps, with ſome remaining ſaponaceous particles.

3. The whole muſt now be evaporated, by a very moderate heat (in a glaſs veſſel, previouſly weighed), until it be perfectly dry. From the difference of weight we ſhall find the abſolute quantity of reſinous and ſaponaceous matter.

4. In order to ſeparate theſe two ſubſtances from each other, the whole ſhould be mixed with four times its weight of vitriolic ether; the retort muſt be provided with a proper neck and receiver, and the mixture ſuffered to digeſt. If a complete ſolution be the reſult, we may conclude that it conſiſted of pure reſin; as, in the contrary caſe, the reſinous matter was mixed with that of ſoap.

5. After the firſt extraction has been performed, the coloured liquor ſhould be poured off, and the reſiduum again digeſted in a new ſupply of vitriolic ether; an operation which ought to be repeated, until the ether newly employed retains its natural colour.

6. If theſe directions be ſtrictly followed, the matter of reſin will be completely diſſolved by the ether, and cleared of the ſaponaceous particles.—Now the different extracts made with ether ſhould be poured into a tubulated

 retort, retort, provided with a receiver, (the weight of which has been previouſly marked) and diſtilled over to dryneſs; thus the ether will be obtained in the receiver, and may ſerve for further experiments. After the retort has become perfectly dry, its increaſe of weight will ſhew the exact quantity of reſin gained in the proceſs.

*On the Separation of Saponaceous Matter.*

The experienced operator on vegetable ſubſtances cannot fail to have obſerved, that the matter of ſoap always forms in them one of the principal conſtituents, ſerving as a vehicle and mean of combination for many other ſubſtances which cannot be ſeparately exhibited. The ſaponaceous ingredient, therefore, requires a principal ſhare of attention in the analyſis of vegetable bodies; for much depends on its accurate ſeparation, if we wiſh to aſcertain the conſtituent parts of vegetables with a due degree of preciſion.

To obtain the matter of ſoap in a ſeparate ſtate, the proceſs to be purſued is exactly ſimilar to that already pointed out for the ſeparation of reſin; for in the preſent caſe, the former ſubſtance ſpontaneouſly remains behind, if the mixture, conſiſting of ſaponaceous and reſinous matter, has been previouſly cleared of the reſinous particles, according to the method already deſcribed. It only requires to be once more diſſolved in water, filtered, and the liquor evaporated to dryneſs, in order to learn the abſolute quantity of ſaponaceous matter; and to be convinced that it contains no admixture of either gummy or mucilaginous particles.

*On the Separation of Saccharine Matter.*

If vegetable ſubſtances be remarkable for their ſweet taſte, we may without heſitation infer, that they contain the ſaccharine principle; but, as this is very frequently blended with gummy, mucilaginous, and particularly with ſaponaceous matter, it ever remains a ſubject of conſiderable difficulty to exhibit the ſugar in a pure and perfect ſtate. Where this valuable ſubſtance is contained in abundance, the operation proves ſometimes ſucceſsful, by extracting ſuch bodies with alcohol, filtering this extract while hot, and afterwards allowing it to grow cold in a quiet place. By this mode of proceeding, real ſugar has frequently been found precipitated in grains.

So far Dr. Hermbstaedt's papers, of which we are in poſſeſſion at preſent. As we are in daily expectation of receiving ſome further accounts from this valuable practical chemiſt; and as Mr. Achard, of Berlin, has lately made very ſucceſsful experiments with the beet root, or the famous *mangel-wurzel*, from which he affirms that he has obtained ſugar of a pure kind, in the proportion of about one pound to fourteen of the freſh root, and this by *an eaſy and cheap proceſs*, we hope to be ſoon enabled to lay before our readers, the *authentic* particulars of theſe new and highly intereſting diſcoveries.

